# Growth defect of domain III glycoprotein B mutants of human cytomegalovirus reverted by compensatory mutations co-localizing in post-fusion conformation

**DOI:** 10.1128/mbio.01812-24

**Published:** 2024-09-24

**Authors:** Madlen Mollik, Andreas Rohorzka, Xiaohan Chen, Barbara Kropff, Lukas Eisler, Büsra Külekci, Elisabeth Puchhammer-Stöckl, Marco Thomas, Irene Görzer

**Affiliations:** 1 Center for Virology, Medical University of Vienna, Vienna, Austria; 2 Virologisches Institut, Klinische und Molekulare Virologie, Friedrich Alexander-Universität Erlangen-Nürnberg, Erlangen, Germany; Columbia University, New York, New York, USA

**Keywords:** human cytomegalovirus, glycoprotein B, glycoprotein O, entry and spread, membrane fusion

## Abstract

Cell entry is a crucial step for a virus to infect a host cell. Human cytomegalovirus utilizes glycoprotein B (gB) to fuse the viral and host cell membranes upon receptor binding of gH/gL-containing complexes. Fusion is mediated by major conformational changes of gB from a metastable pre-fusion to a stable post-fusion state whereby the central trimeric coiled-coils, formed by domain (Dom)III α helices, remain structurally nearly unchanged. To better understand the role of the stable core, we individually introduced three potentially helix-breaking or one disulfide bond-breaking mutation in the DIII α3 to study different aspects of the viral behavior upon long-term culturing. Two of the three helix-breaking mutations, gB_Y494P and gB_I495P, were lethal for the virus in either fibroblasts or epithelial cells. The third substitution, gB_G493P, on the other hand, displayed a delayed replication and spread, which was more pronounced in epithelial cells, hinting at an impaired fusion. Interestingly, the disulfide bond-breaker mutation, gB_C507S, performed strikingly differently in the two cell types - lethal in epithelial cells and an atypical phenotype in fibroblasts, respectively. Replication curve analyses paired with the infection efficiency, the spread morphology, and the cell–cell fusogenicity suggest a dysregulated fusion process, which could be reverted by second-site mutations mapping predominantly to gB DomV. Our findings underline the functional importance of a stable DomIII core for a well-regulated DomV rearrangement during fusion.

**IMPORTANCE:**

Human cytomegalovirus (HCMV) can establish a lifelong infection. In most people, the infection follows an asymptomatic course; however, it is a major cause of morbidity and mortality in immunocompromised patients or neonates. HCMV has a very broad cell tropism, ranging from fibroblasts to epi- and endothelial cells. The virus uses different entry pathways utilizing the core fusion machinery consisting of glycoprotein complexes gH/gL and glycoprotein B (gB). The fusion protein gB undergoes fundamental rearrangements from a metastable pre-fusion to a stable post-fusion conformation. Here, we characterized the viral behavior after the introduction of four single-point mutations in the gB central core. These led to various cell type-specific atypical phenotypes and the emergence of compensatory mutations, demonstrating an important interaction between domains III and V. We provide a new basis for the development of a structurally and functionally altered gB, which can further serve as a tool for drug and vaccine development.

## INTRODUCTION

Glycoprotein B (gB) is the highly conserved class III fusogen of herpesviruses (1). The three-dimensional conformations of pre- and post-fusion gB structures of different herpesviruses are well conserved (2) despite limited sequence similarity. Human cytomegalovirus (HCMV) UL55 encodes the 907-amino acid (aa) gB polypeptide (numbering according to TB40/E). Processing by proteolytic cleavage at the furin site (3, 4) results in an amino-terminal (~116 kDa) and carboxy-terminal (~58 kDa) fragment, held together by two intramolecular disulfide bonds between cysteine residues C94 and C551 in domain (Dom)IV, as well as C111 and C507 in DomIII (3). gB is a membrane-bound homotrimer whose trimeric coiled-coils, also defined as the central core, are formed by the α3 helices of DomIII, centered on the threefold axis of the ectodomain generating the trimerization contacts of the protomers (1, 5–7). While a comparison of gB pre- and post-fusion structures reveals major structural rearrangements during the transition process, the α3 helices of the protein seem to remain nearly unchanged (6) indicating the importance of this core stabilization for fusion itself.

Herpesvirus gB is non-autonomously fusogenic (8, 9). Instead, it requires activation by gH/gL-based glycoprotein complexes (10), which in HCMV are further linked to the accessory proteins glycoprotein O (gO) (trimer) or UL128, UL130, and UL131A (pentamer) (11). A current model of HCMV cell entry suggests that either the trimer, the pentamer, or both interact with pre-fusion gB while binding of a gH/gL complex to the host cell receptor brings the viral and cellular membrane to close proximity for the gB fusion loops to insert into the membrane (6, 12). Here, gB undergoes a major conformational change from a metastable pre-fusion to a stable post-fusion conformation resulting in the merging of the two membranes and the release of the viral content into the cell (1, 6). Fusion at the plasma membrane of fibroblasts is pH-independent (13) and in the endosome of epithelial, endothelial, and myeloid cells pH-dependent (14). After entry, a strict cell-to-cell spread was observed in clinical isolate culturing (15), the likely mode of dissemination in the human host (16, 17). Cell culture-adapted strains have at least partially lost this spread mode mainly due to mutations in the pentamer or RL13 locus (18, 19). gB and gH/gL complexes are required for both, the cell-free and the cell-to-cell spread (20), as small inhibitors directed toward gO (21, 22) or gB (23) are able to inhibit both spread modes.

The most diverse genes of the core fusion machinery are gB, gH, and gO expressing five, two, and eight distinct genotypes (GTs), respectively (24). Those genotypes are widely distributed in clinical isolates (25–27), existing in numerous combinations (28, 29) but without a clear association to different disease severities. On the single-gene level, it appears that gB-mediated fusion can be differentially modulated by variants of individual components. As recently reported, a unique variant of gB, 275Y of AD196 strain and 585G of VR1814 strain, contributes to increased fusogenicity due to inherently hyperfusogenic gB or altered interaction with a gH/gL complex (30). Furthermore, the gB genotype variability at the furin site influences the cleavage efficiency and probably the gB fusion capacity (4). gH polymorphism contributes to the generation of gH genotype-specific antibodies (31), and gO polymorphism alters the neutralizing capacity of gH and gH/gL-specific monoclonal antibodies (32, 33) and may influence the trimer-to-pentamer ratio (34), an important determinant for the mode of spread (35).

In the present study, we focused on the large α3 helix of DomIII forming the stable central core of gB. We selected four aa residues, G493, Y494, I495, and C507, for single-point mutagenesis in the background of TB40-BAC4-derived strains with three different gO genotypic sequences, GT1c, GT3, and GT1c3 (36). Helix-breaking mutations to proline (G493P, Y494P, and I495P) may contribute to a stabilization of gB in the pre-fusion conformation as recently shown for HSV-1 and VZV (37, 38). The disulfide bond-breaking mutation (C507S) may affect gB protomer coherence upon furin cleavage important for the structural stability of the homotrimer (3). Long-term culturing of bacterial artificial chromosome (BAC)-derived gB mutants demonstrated mutation- and cell type-dependent growth alterations, however with no prominent influence of the gO genotypic form. Second-site mutations in gB emerged exclusively in fibroblast-derived gB_C507S mutants, predominantly located in DomV, and were associated with a rescue of the infection capability on both cell types.

## RESULTS

### gB mutants reveal mutation- and cell type-dependent growth impairment

**Fig 1:**
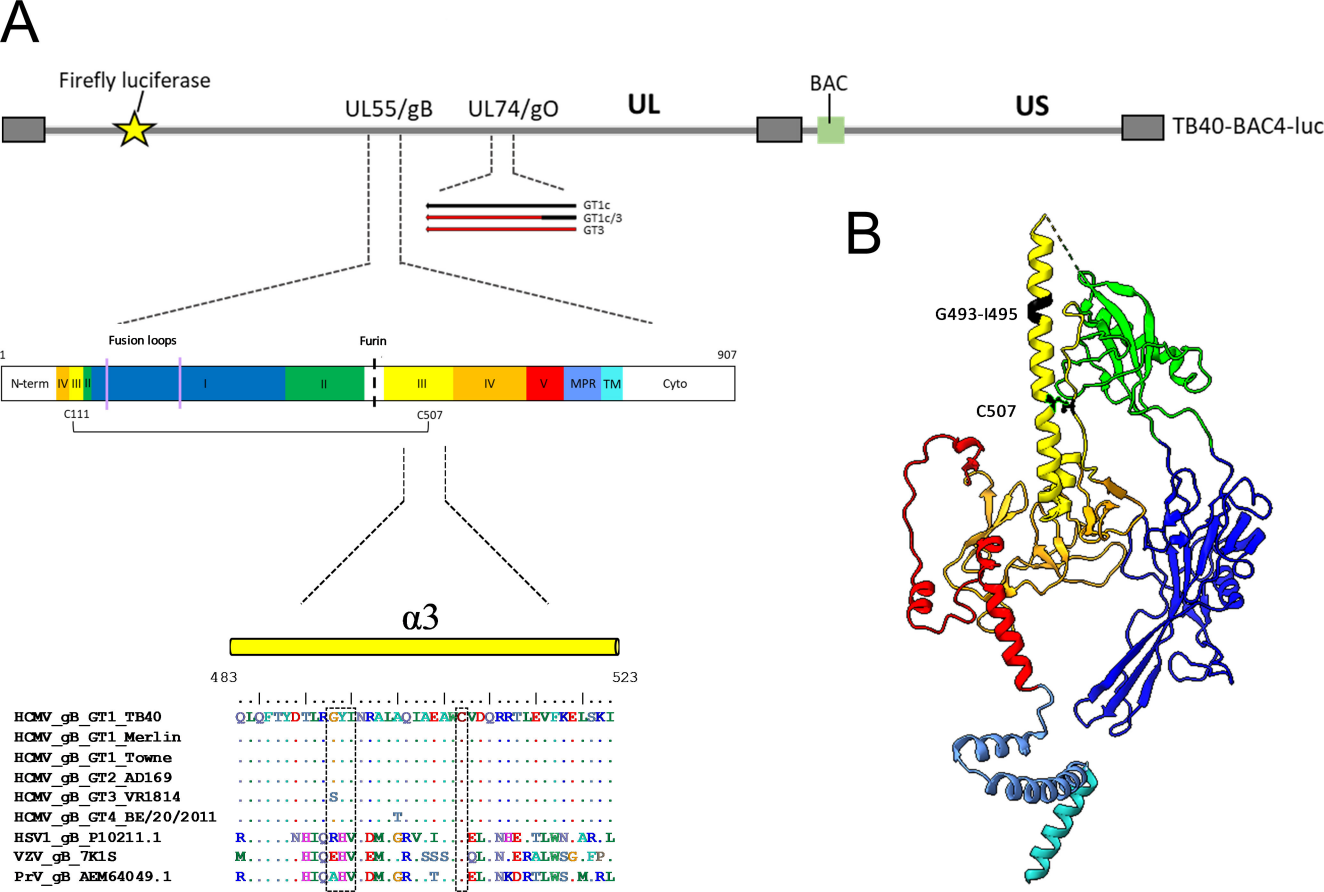
Schematic of TB40-BAC4-luc DNA and protomer gB pre-fusion structure. (**A**) Illustration of TB40-BAC4-luc DNA with main genome characteristics used for the transfection and reconstitution in fibroblasts and epithelial cells. Diagram of gB encoded by UL55 displaying the C111-C507 disulfide bond; fusion loops are indicated with a purple line, the furin cleavage site is marked with a dotted black line and the five structural gB DomI–V, MPR, and TM in color. Enlarged the sequence alignment of the α3 helix with representative sequences of HCMV, HSV-1, VZV, and PrV as indicated. Location of the mutations are depicted by dotted black boxes in the alignment. (**B**) Protomer structure (PDB: 7KDP). Mutation sites G493-I495 and C507, forming a disulfide bond with C111, are marked in black. gB domains are colored as follows: DomI (dark blue), DomII (green), DomIII (yellow), DomIV (orange), DomV (red), the membrane proximal region (MPR, blue), and the transmembrane domain (TM, turquoise) created with UCSF ChimeraX [61]. GT, genotype; HSV-1, herpes simplex virus 1; VZV, varicella zoster virus; PrV, pseudorabies virus; N-term, N-terminal region; Cyto, cytoplasmic tail.

**Fig 2:**
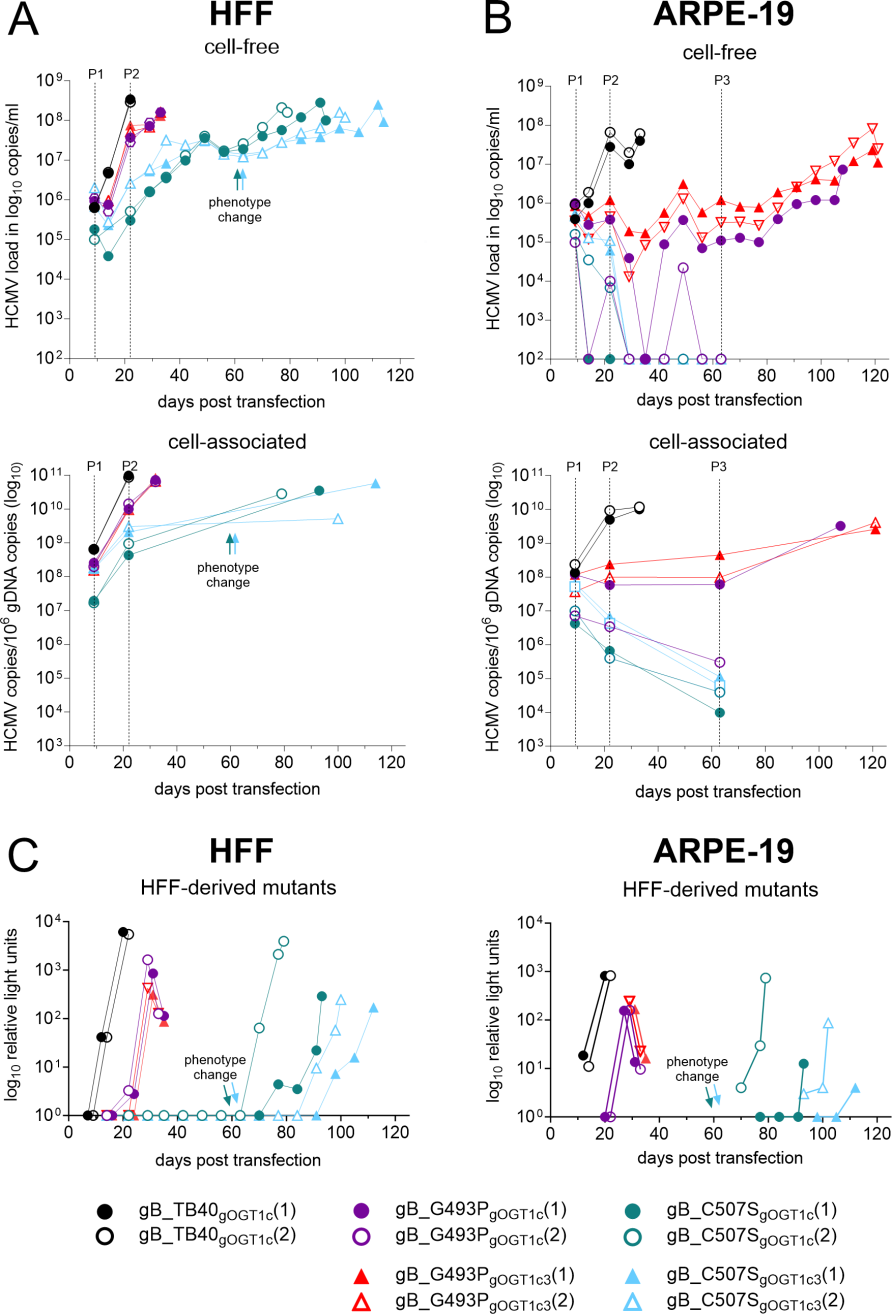
Viral replication and infectivity differences among gB mutants independent of gO genotypes. (**A and B**) HFF or ARPE-19 were individually transfected in duplicates with the respective TB40-BAC-luc DNA and long-term cultured. During reconstitution, cell-free samples were taken from the supernatant once a week during medium change, and cell-associated samples were taken during passaging (P1–P3) as indicated. (**A** and B) Top panel: viral DNA released into the supernatant of transfected HFF (**A**) or ARPE-19 cells (**B**) with the parental gB_TB40_gOGT1c_ in black, gB_G493P mutants in purple and red, and gB_C507S mutants in petrol and light blue, each in gOGT1c and gOGT1c3 background, respectively. (**A** and B) Bottom panel: viral DNA load in cell-associated samples of transfected HFF (**A**) or ARPE-19 cells (**B**) normalized to the genomic DNA. (**C**) Subsequent to the harvest of all gB clones, the infection efficiency of cell-free viruses was tested in parallel through a luciferase assay. After infection, HFFs were incubated for 48 h and ARPE-19 cells for 72 h before the determination of relative light units (RLUs) in the cell lysates as the read-out of infection efficiency. Positive samples on HFFs were then tested on ARPE-19 cells (right). P, passaging; gDNA, genomic DNA.

The four-point mutations in the α3 helix of gB DomIII were individually introduced into the TB40-BAC4-luc strain (Fig. 1; Table 1). Two independent transfections and their subsequent analyses were performed in parallel under the same conditions in both human foreskin fibroblasts (HFFs) and adult retinal pigment epithelial cells (ARPE-19), with gB mutants harboring the gO background GT1c or GT1c3, respectively. In an additional experiment, gB mutants with gO GT3 background were mainly analyzed on HFFs. Passaging was performed after 9 and 22 days post-transfection (dpt) in HFFs and after 9, 22, and 63 dpt in ARPE-19 cells to follow the evolution of the morphology of the infectious foci, which might otherwise be masked by multiple passaging steps leading to the extensive spreading of the infected cells throughout the cell monolayer. Viral DNA was measured within the cell (cell-associated) and in the culture supernatant (cell-free) (Fig. 2A and B) over a time period of up to 121 dpt until a cytopathic effect (CPE) ≥80% was seen in HFFs and ≥50% in ARPE-19 cells (Table S1a).

**TABLE 1:** Transfection efficiency of parental and gB mutated viral genomes based on TB40-BAC4-luc into fibroblasts and epithelial cells^*a*^^,^^*b*^

BAC-derived viruses	gB mutation^*c*^	gO origin; genotype	HFF				ARPE-19			
			Visible CPE	RLU at 9 dpt	RLU at 22 dpt	RLU at harvest	Visible CPE	RLU at 9 dpt	RLU at 22 dpt	RLU at 63 dpt or harvest
gB_TB40_gOGT1c_		TB40; GT1c	Yes	+	++	+++	Yes	+	++	+++
gB_G493P_gOGT1c_	G493P	TB40; GT1c	Yes	+	++	+++	Yes	+	+	+++^*d*^
gB_Y494P_gOGT1c_	Y494P	TB40; GT1c	No	+			No	+	−	−
gB_I495P_gOGT1c_	I495P	TB40; GT1c	No	+	++		No	+	−	−
gB_C507S_gOGT1c_	C507S	TB40; GT1c	Yes	+	++	+++	No	+	++	−
gB_TB40_gOGT1c3_		TB40/HAN16; GT1c3	Yes	+	++	+++	Yes	+	++	+++
gB_G493P_gOGT1c3_	G493P	TB40/HAN16; GT1c3	Yes	+	++	+++	Yes	+	++	+++
gB_Y494P_gOGT1c3_	Y494P	TB40/HAN16; GT1c3	No	+			No	+		
gB_C507S_gOGT1c3_	C507S	TB40/HAN16; GT1c3	Yes	+	++	+++	No	+	−	−
gB_TB40_gOGT3_		HAN16; GT3	Yes	+						
gB_G493P_gOGT3_	G493P	HAN16; GT3	Yes	+						
gB_Y494P_gOGT3_	Y494P	HAN16; GT3	No	+			No	+	−	−
gB_I495P_gOGT3_	I495P	HAN16; GT3	No	+			No	+	−	−
gB_C507S_gOGT3_	C507S	HAN16; GT3	Yes	+						

^*a*^ ^+^RLU positive; ^++^increase in RLU compared to the first measurement; ^+++^increase in RLU compared to the second measurement; ^−^RLU negative.

^*b*^ GT, genotype; RLU, relative light units; TB40-BAC4 (GenBank: EF999921.1); HAN 16 (GenBank: JX512204.1).

^*c*^ Numbering according to TB40-BAC4.

^*d*^ RLU measurement of a single transfection, all others from the mean of duplicate transfections.

First, gB_Y494P and gB_I495P mutants, across the different gO genotypical backgrounds, failed to demonstrate any detectable increase in viral DNA in either cell type. This was observed despite the verification of BAC-DNA sequences prior to the transfection and repeated successful transfections of BAC-DNA (Table 1). Consequently, no further culturing experiments were conducted.

Second, the replication curves (as assessed by cell-associated and cell-free viral DNA) of gB_G493P_gOGT1c_ and gB_G493P _gOGT1c3_ displayed a similarly slight delayed increase in the viral DNA load in HFFs when compared to the parental strain (Fig. 2A). Parental and gB_G493P viruses were harvested at 22 and 29 dpt, respectively, with both reaching a CPE of 100%. In an independent experiment, gB_G493P_gOGT3_ showed a more severe replication delay but displayed similarly high viral DNA loads as the parental strain at harvest time (Fig. S1). In ARPE-19 cells, viral loads of gB_G493P_gOGT1c_ and gB_G493P_gOGT1c3_ remained almost at the same level and increased up to 100-fold after passage 3 until harvest (Fig. 2B). Notably, gB_G493P_gOGT1c_ clone 2 failed to establish long-term replication probably due to the low number of initial post-transfection foci (2, 3) getting lost after passaging (Fig. 2B).

Third, gB_C507S already displayed strongly delayed replication kinetics in HFFs (Fig. 2A; Fig. S1) and even failed to replicate in ARPE-19 cells (Fig. 2B) despite successful transfection (Table 1). In the cell-associated fraction of HFFs, the viral DNA of gB_C507S_gOGT1c_ and gB_C507S_gOGT1c3_ readily increased from 9 to 22 dpt comparable to gB_G493P (mean of 1.4 versus 1.7 log_10_ copies/mL) and slightly lower than the parental strains (mean of 2.2 log_10_ copies/mL) (Fig. S2). The accompanied release of viral DNA (from 14 to 22 dpt) was significantly lower in gB_C507S (mean of 1.1 log_10_ copies/mL) than in parental strains (mean of 2.5 log_10_ copies/mL). Moreover, after a steady release of viral DNA for up to around 40 dpt, a stagnation period for 14–21 days set in, followed by an increase in viral load, which coincided with a change in the morphology of the foci, termed phenotype change, as described below. Eventually >10^8^ copies/mL were reached at harvest (Fig. 2A). gB_C507S_gOGT3_ displayed an even longer stagnation period (~30 days), thus was harvested after 70 dpt and further propagated for another 30–50 days (Fig. S1). Prolonged low-level replication may allow for the emergence of additional mutations with the potential to rescue the growth defect.

In summary, replication curve analyses upon transfection of BAC-DNA revealed that Y494P and I495P were crucial for viral replication while G493P and C507S mutants revealed striking growth differences in various cell types but without an obvious influence of the corresponding gO genotypes.

### Fibroblast-derived gB_C507S are infectious for fibroblasts and epithelial cells after phenotype change

Next, we determined the infection efficiency of the fibroblast- and epithelial cell-derived viruses (parental strain, gB_G493P, and gB_C507S), which were regularly collected during the transfection period (Fig. 2A and B). For this, HFF and ARPE-19 cells were infected with the cell-free viruses, and luciferase expression was monitored as relative light units (RLUs) in cell lysates 2–3 days after infection, respectively. As shown in Fig. 2C, the initial detection of infectious fibroblast-released virus was earliest for the parental strain, followed by gB_G493P, and was substantially delayed for gB_C507S on either cell type. Strikingly, gB_C507S gained the ability to infect not only HFF but also ARPE-19 cells only after the phenotype change occurred (Fig. 2C) and despite the lack of replication upon the transfection of mutant BAC-DNA in ARPE-19 cells (Fig. 2B). The fibroblast-released gB mutants had a lower infection capacity for ARPE-19 cells than for HFFs similar as the parental strain (Fig. S3B). The infectivity of epithelial cell-released viruses was low for the parental strain and undetectable for the gB mutants in either cell type (Fig. S3A).

**Fig 3:**
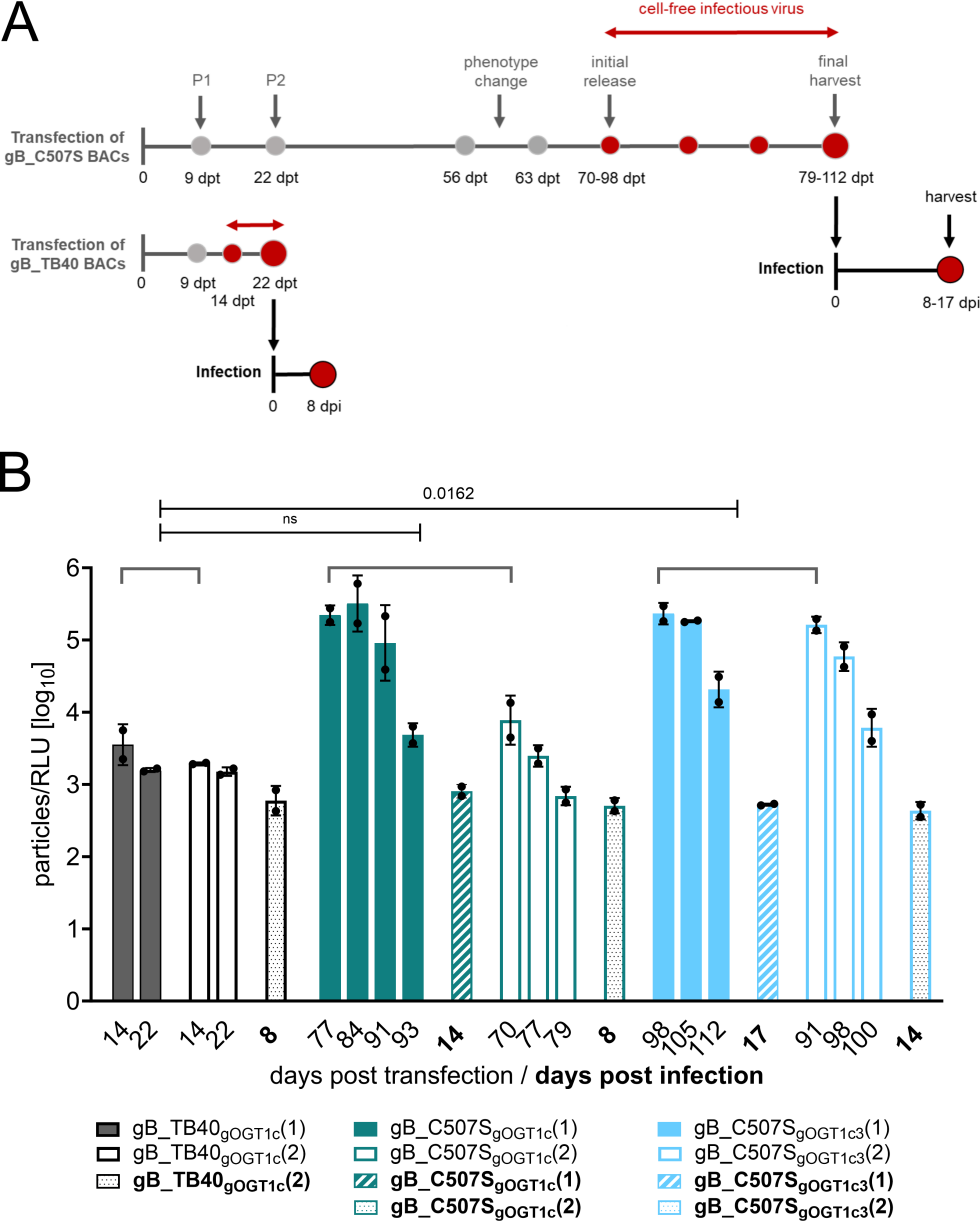
Increase in cell-free infectivity of gB_C507S mutant viral particles late after transfection and further propagation. (**A**) Experimental scheme showing the transfection and infection period with the time points indicating the main characteristics: passaging (**P1 and P2**) and pre- and post-phenotype change of C507S mutants in gray dots and initial and further release of infectious virus into supernatant (cell-free) until harvest post-transfection and post-infection in red dots. (**B**) Infectious cell-free parental and gB_C507S virus stocks at the indicated dpt and dpi were investigated for (i) the number of encapsidated genome particles and (ii) the infection efficiency. Encapsidated genome copy numbers were determined by HCMV-specific quantitative PCR after DNase I treatment. Infection capacity was assessed in parallel 48 h after HFF infection by measuring the RLUs in cell lysates. Calculated particle-to-RLU ratios in log_10_ are plotted for parental and gB_C507S mutant clones 1 (black and colored columns) and 2 (open columns) post-transfection and post-infection (columns with patterns). Colors for the mutant clones are the same as in Fig. 2. Columns indicate the mean. Error bars are the standard deviation. A comparison of the medians from two clones in each group (in duplicate) as indicated by the brackets was performed using the non-parametric Kruskal–Wallis test and Dunn’s *post hoc* test for multiple comparisons. ns, not statistically significant (*P* > 0.05).

The delay in the release of fibroblast-derived infectious gB_C507S virus after transfection led us to determine the specific infectivity (ratio of the number of virus particles to RLU). For this, cell-free virions in infectious supernatant stocks of two clones each of the parental strain gB_TB40_gOGT1c_ and the gB_C507S mutant (Fig. 2C and 3A) were analyzed by quantitative PCR for encapsidated viral genomes and by luciferase expression in HFFs for infection efficiency. At the time of initial release, the mean particle-to-RLU ratio (both clones combined) was 16-fold and 74-fold higher for gB_C507S_gOGT1c_ and gB_C507S_gOGT1c3_, respectively, compared to the parental strain, reaching statistical significance for gB_C507S_gOGT1c3_ (Fig. 3B). The specific infectivity of all C507S mutants improved over time, as displayed by a decrease in the particle-to-RLU ratio, and eventually became as infectious as the parental strain when subsequently propagated on HFFs for an additional 8–17 days post-infection (dpi) (Fig. 3A and B).

### Morphology of the cytopathic effect depends on the gB point mutation

**Fig 4:**
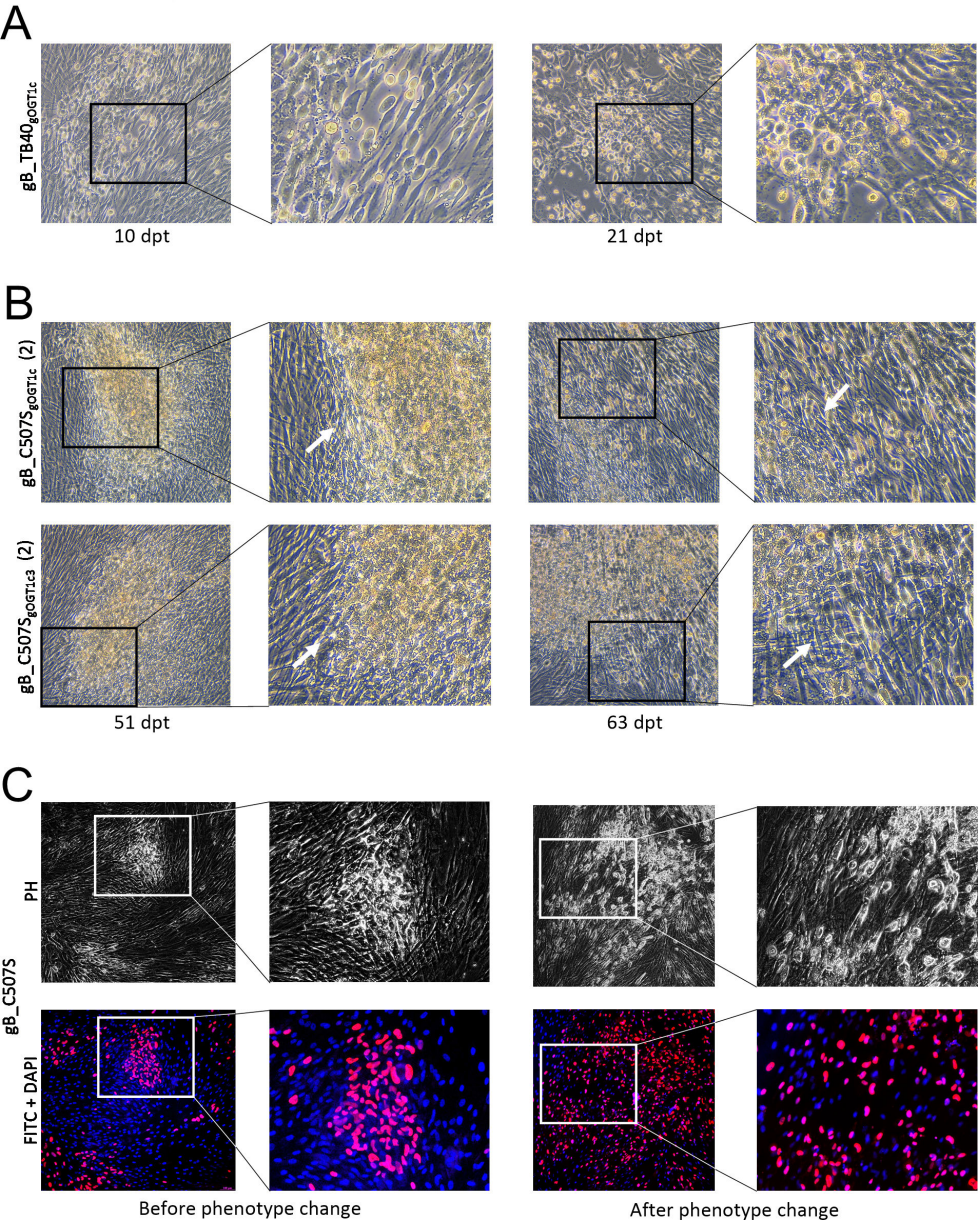
Morphology change of gB_C507S mutant foci on fibroblasts during long-term culturing. (**A and B**) After transfection of HFFs with the parental (**A**) and the gB mutant TB40-BAC-luc DNA clones (**B**) as indicated, light microscopy (Leica) pictures were regularly taken during long-term culturing indicated with dpt. Representative images (10×) of cytopathic effects of parental strain (**A**) compared to gB_C507S mutants (**B**), displaying highly dense foci at 51 dpt and beginning to change the phenotype to an evenly spread morphology at around 63 dpt. The black boxes highlight the areas shown on the right. White arrows indicate the definable (left panels) and the indistinct (right panels) borders of the foci to the surrounding cell monolayer. (**C**) gB_C507S mutants harvested prior to (left panels) and after (right panels) the phenotype change were subjected to immediate early (IE) and DAPI staining 22 days after HFF infection (dpi) when the characteristic phenotype was present. The white boxes highlight the areas shown on the right. Representative plaques of the focal growth and the even spread are shown in 10×. (1), clone 1; (2), clone 2; PH, phase contrast; FITC, fluorescein isothiocyanate; DAPI, 4′,6-diamidino-2-phenylindole.

The CPE of parental TB40-BAC4-luc virus is characterized by an even, supernatant-driven spread in HFFs while it is more restricted in ARPE-19 cells (39, 40). Here, the CPE was visually inspected every 3–4 days by light microscopy over the whole time course of the experiment. The morphologies markedly differed among the gB mutants and between the two cell types. In HFFs, all gB_G493P mutants except gB_G493P_gOGT3_ clone 1 showed a parental strain-like CPE with an even spread of >90% CPE at harvest (Fig. S4A and B and S5A and B). gB_G493P_gOGT3_ clone 1 presented a partially focal phenotype, which appeared after 38 dpt and remained until harvest (Fig. S5B). In ARPE-19 cells, three of the four tested gB_G493P clones showed CPE similarity to the parental strain while gB_G493P_gOGT1c3_ clone 2 displayed one highly dense focus in addition to the parental-like CPE, which prevailed after passaging until 121 dpt (Fig. S4B). In contrast to gB_G493P, all clones of gB_C507S displayed a similarly aberrant spread pattern on HFFs (Fig. 4B; Fig. S4C and S5C). After transfection, a strictly focal CPE was formed, and the foci appeared as highly dense accumulations of cells showing a definable border to the surrounding uninfected cell monolayer (Fig. 4B and C). From around day 63 onward, the borders of the individual foci became indistinct, and the virus began to spread throughout the monolayer (Fig. 4B and C) resembling the parental strain CPE (Fig. 4A). At harvest, a combination of both led to a >80% CPE (Fig. S4C and S5C). Of note, pictures from parental strain CPE were chosen from earlier time points due to the rapid virus spread. Overall, a variety of different spread phenotypes ranging from cell-associated to cell-free patterns and a switch thereof could be observed during long-term culturing with no clear indication of a differential influence of the analyzed gO genotypes.

### gB_C507S rather than gB_G493P acquires second-site mutations in gB post-transfection

**Fig 5:**
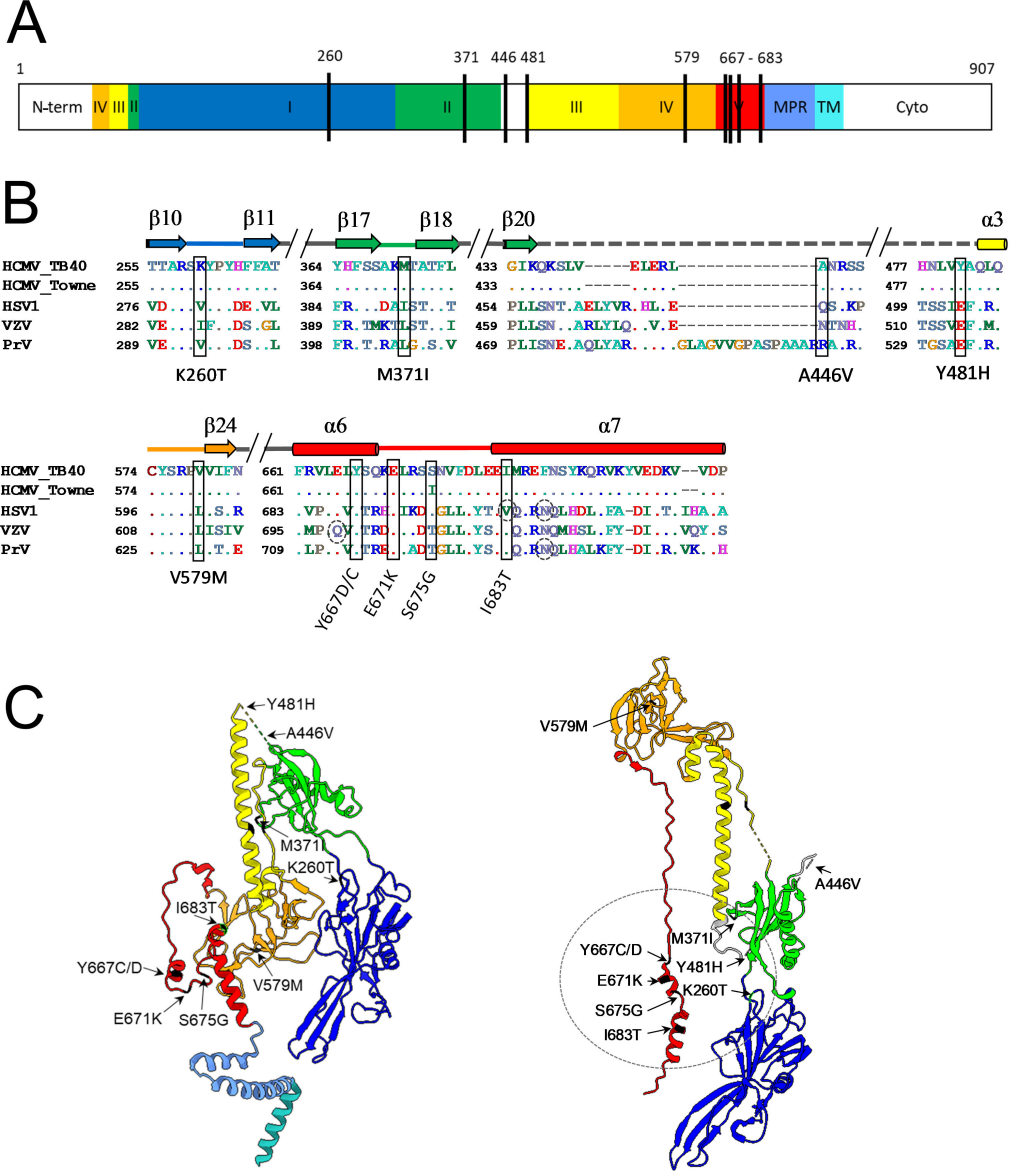
Co-localization of second-site mutations of gB_C507S mutants in gB post-fusion structure. (**A**) Schematic diagram of linear gB marked with second-site mutations as black lines that emerged in all gB_C507S clones during the post-transfection and post-infection period (see Table 2). (**B**) Amino acid alignments of gB from two HCMV strains (TB40 and Towne) and three alphaherpesviruses (HSV-1, VZV, and PrV) as indicated with the homologous sites of the second-site mutations highlighted with black boxes. Respective substitutions are shown below. Broken circles indicate the residues in HSV-1 and PrV for which fusion mutants have been described (41–43). (C) Protomer structure of gB with C507 in α3 helix marked in black. Acquired second-site mutations are highlighted in pre-fusion (left; PDB: 7KDP) and post-fusion protomer (right; PDB: 5C6T) with the majority of those located in the encircled post-fusion region. Created with UCSF ChimeraX [61]. Domains in all depictions are colored as in Fig. 1. α, alpha helix; β, beta sheet.

The distinct CPE patterns prompted us to investigate the HCMV genome for the evolution of reversion and/or second-site mutations after long-term culturing post-transfection. Therefore, whole genome sequencing (WGS) was performed using the cell-associated and/or cell-free viral DNA collected at harvest without any further enrichment. As listed in Table S1a and b, this included eight clones from parental strains (HFF, *n* = 6; ARPE-19, *n* = 2) and 15 gB mutant clones (HFF, *n* = 12; ARPE-19, *n* = 3). All gB mutants maintained the introduced point mutations, while no mutations at all were observed for the parental strains in both cell types (Table S1a). Seven out of 15 gB mutant clones acquired additional mutations at different genomic locations and various frequencies, accompanied by changes in the CPE morphology (Table S1b). gB_G493P_gOGT3_ clone 1 developed a single-nucleotide polymorphism in HFFs leading to UL76_L131I and UL77_S324Y substitutions at a frequency of ~80%. gB_G493P_gOGT1c3_ clone 2 acquired a UL131A_T101S mutation in ARPE-19 cells at almost 100% abundance. No further genomic mutations were observed for other gB_G493P clones in both cell types, and no second-site mutations developed in gB. This is in stark contrast to the six fibroblast-derived gB_C507S clones, of which five developed at least one additional mutation in gB during the post-transfection period (Table 2; Table S1b). These substitutions were found at various frequencies (≤71%) and different regions, yet particularly concentrated in DomV (Fig. 5A through C). Furthermore, gB_C507S_gOGT1c_ clone 2 and gB_C507S_gOGT1c3_ clone 2 each acquired genomic mutations in UL48_A1684V and UL148_D247A, respectively. Together, the emergence of the additional mutations seems to be associated with the type of initially introduced mutation and with the appearance of a phenotype switch rather than with a certain gO genotype.

**TABLE 2:** Appearance and frequency of occurrence of second-site gB mutations in fibroblast-derived gB_C507S mutants detectable in cell-free and cell-associated fraction during long-term culturing after transfection and after infection of reconstituted cell-free viruses^*a*^

BAC-derived viruses (clone)	Second-site gB mutations after transfection (frequency in %; dpt)					Second-site gB mutations after infection (frequency in %; dpi)	
	Amp-CF	Amp-CF	Amp-CF^*b*^	Amp/WGS-CF	WGS-CA	Amp-CF	WGS-CA
	56 dpt	63 dpt	70–98 dpt	At harvest	At harvest	At harvest	At harvest
gB_C507S_gOGT1c_(1)	–	A446V (9%)	A446V (45%; 77)	A446V (69%; 93)	A446V (56%; 93)	A446V (88%; 14)	A446V (88%; 14)
gB_C507S_gOGT1c_(2)	–	–	Y667D (26%; 70)	Y667D (71%; 79)	Y667D (64%; 79)	Y667D (73%; 8)	Y667D (77%; 14^*c*^)
							
gB_C507S_gOGT1c3_(1)	–	–	–	K260T (15%; 112)	K260T (12%; 112)	K260T (36%; 17)	K260T (40%; 17)
			Y667C (24%; 98)	Y667C (43%; 112)	Y667C (37%; 112)	Y667C (57%; 17)	Y667C (57%; 17)
gB_C507S_gOGT1c3_(2)	–	–	V579M (8%; 91)	V579M (15%; 100)	V579M (10%; 100)	V579M (40%; 14)	V579M (44%;14)
			E671K (48%; 91)	E671K (43%; 100)	E671K (34%; 100)	E671K (45%; 14)	E671K (44%; 14)
gB_C507S_gOGT3_(1)	nd	nd	nd	E671K (18%; 69)	–	nd	E671K (82%; 50)
gB_C507S_gOGT3_(2)	nd	nd	nd	–	–	nd	M371I (66%; 30)
							Y481H (11%; 30)
							S675G (7%; 30)
							I683T (9%; 30)

^*a*^ Amp, amplicon sequencing; dpt, days post-transfection and dpi, days post-infection; –, not detected; nd, not determined; CF, cell-free; CA, cell-associated.

^*b*^ First time point after transfection when infectious cell-free viruses are detectable.

^*c*^ Monolayer largely destroyed; amino acid numbering according to gB of TB40-BAC4.

### Detection of second-site gB mutations shortly after the phenotype change

Next, we asked whether the additional gB mutations of fibroblast-derived gB_C507S found at harvest were already detectable around the observed phenotype change post-transfection and how they develop after subsequent propagation on fibroblasts (post-infection) (Fig. 3A). Collected supernatants prior to (56 dpt) and shortly after (63 dpt) the CPE change and at the initial detection of infectious viruses (70–98 dpt) were available for four clones (gB_C507S_gOGT1c_ and gB_C507S_gOGT1c3_) and from all six clones after HFF propagation (Table 2). Isolated viral DNA was subjected to WGS and/or gB long-range PCR and in-depth sequencing. No second-site gB mutations were detectable before the phenotype change, while in gB_C507S_gOGT1c_ clone 1, A446V appeared shortly afterward at a frequency of 9%, and all others displayed second-site gB mutations when infectious virions were released (Table 2). The abundance of the additional mutations increased until harvest and further during propagation. Concurrently, the particle-to-RLU ratio decreased rendering the mutants more infectious (Fig. 3B). This inverse relationship strongly suggests that the emergence of second-site mutations in gB is responsible for the observed phenotype change.

### Second-site gB domain V mutations compensate for the fusion defect of gB_C507S

**Fig 6:**
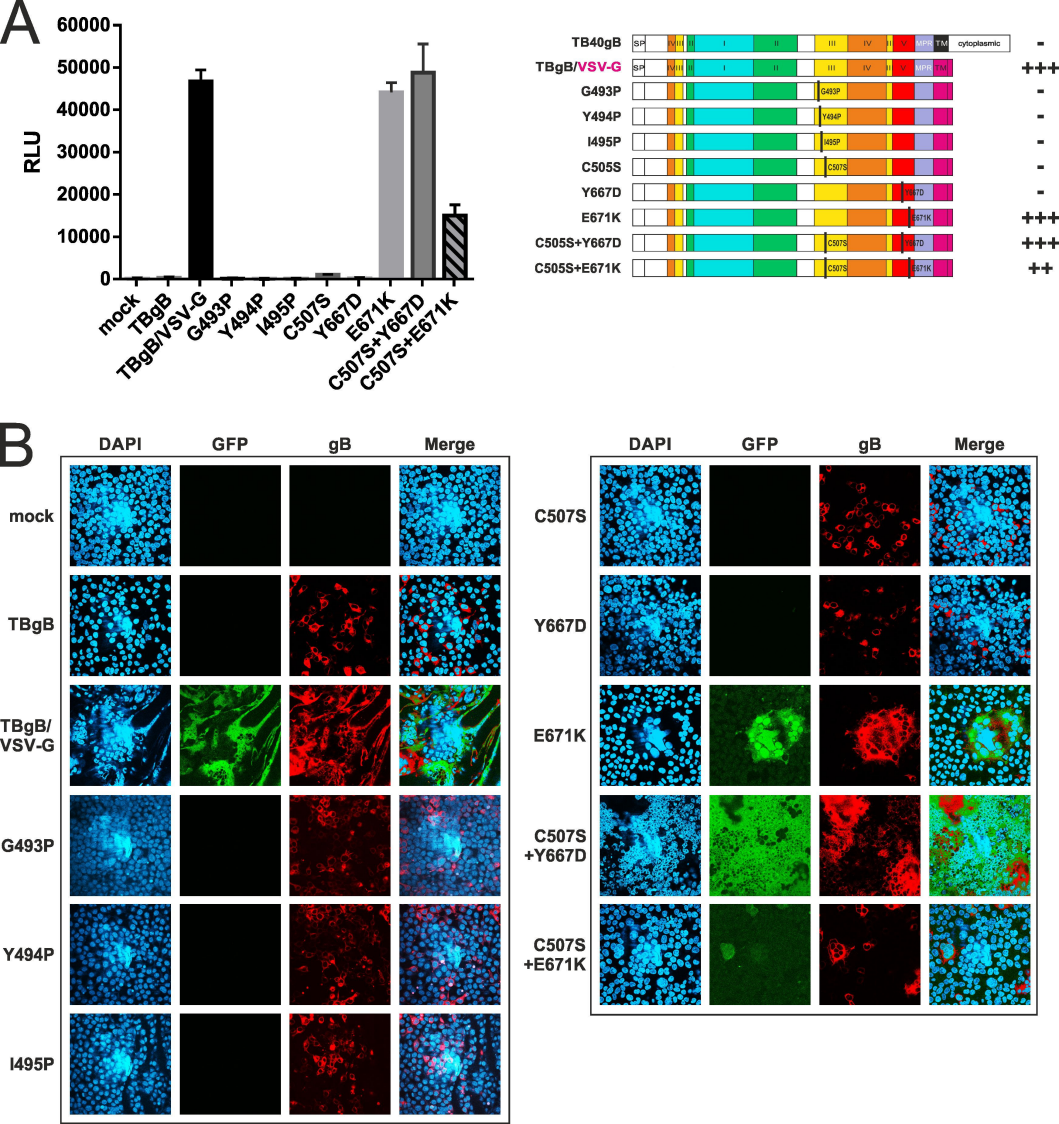
Compensation of the cell–cell fusion defect of gB_C507S by second-site mutations in gB domain V. (**A**) The 293T-DSP-mix cells were transiently transfected with an empty vector (mock), wild-type full-length TB40gB (TBgB), the intrinsically fusion-competent TBgB/VSV-G without mutations, or any of the indicated mutated versions thereof. Cell–cell fusions were quantified via bioluminescence, given in RLUs depicted as mean values of biological triplicates ± standard deviations. (**B**) Immunofluorescence analyses of 293T-DSP-mix cells were performed 3 days after transfection with the same expression plasmids as used in (A). After fixation, cell nuclei were visualized using DAPI and the GFP signal of the reconstituted DSP-reporter protein upon cell–cell fusion. As a control, gB expression was determined by anti-gB MAb 27-287. Representative confocal laser scanning microscopy images are given. TBgB/VSV-G, chimeric version of gB (aa 1–750 of strain TB40E; ABV71586.1) with vesicular stomatitis virus G protein (VSV-G) transmembrane and cytoplasmic tail as previously described (44); mock, empty pcDNA3 vector; MAb, monoclonal antibody.

Finally, we took advantage of a recently established dual split assay (DSP) based on the complementation of green fluorescence protein (GFP) and luciferase to quantify the fusogenic activity of G493P, Y494P, and I495P as well as C507S, Y667D, and E671K either alone or in combination. We selectively chose the two Y667D and E671K second-site mutations for further study, as both are located in DomV and were identified as unique mutations, and the affected residues were independently mutated in separate experiments (Table 2). To assess their impact on cell–cell fusion, the respective mutations were introduced into the intrinsically fusion-active TBgB/VSV-G variant, which facilitates cell–cell fusion in the absence of gH/gL, as previously described (44). After transfection of the respective plasmids into 293T-DSP-mix cells, fusogenic activity was quantified through bioluminescence (Fig. 6A) as well as reconstituted GFP fluorescence (Fig. 6B). While all mutated forms of TBgB/VSV-G were expressed, no fusogenicity was measured for the three helix-breaking proline mutants and not for C507S or Y667D, hence suggesting a functional defect. The combination of either of the two DomV mutations Y667D and E671K with C507S restored the fusogenic potential of this chimeric form of gB. However, C507+E671K was less fusogenic and reached only about 30% of the fusion capacity of unmutated TBgB/VSV-G. Notably, E671K alone did not affect fusogenicity compared to the unmutated gB/VSV-G chimera. Together, these results clearly demonstrate that both of these second-site mutations in gB compensate for the fusion defect of C507S.

## DISCUSSION

Using targeted mutagenesis on BAC-derived viruses, we demonstrate that single-point mutations in the central α helix of DomIII HCMV gB cause severely distinctive growth defects in fibroblasts and epithelial cells, reinforcing the mechanistic differences of gB-mediated fusion between these two cell types (45). *In vitro* evolution upon long-term culturing reveals compensatory gB mutations that are predominantly concentrated in DomV corroborating a cooperative regulatory role of DomIII and DomV during fusion.

The most exceptional phenotype was displayed by gB_C507S on fibroblasts. Each α3 of the three gB protomers forms a coiled-coil structure leading to a stable central structure of the homotrimer, which stays nearly unchanged during the pre- to post-fusion transition (6). Further, C507 forms a disulfide bond with C111 to maintain the association of the N- and C-terminal gB fragments upon furin cleavage (3, 5). We hypothesize that the disruption of this disulfide bond may weaken the stability, thereby affecting the fusion process. While in an experimental cell–cell fusion assay with gB expression constructs C507S abolishes gB fusogenicity in the absence of gH/gL-containing complexes, our virus data show that gB_C507S display tightly localized foci similar to a cell-to-cell spread in fibroblasts, which contrasts the evenly distributed cell-free spread of the parental TB40-BAC4-luc strain (Fig. 4A and B) (39, 40). Despite this obvious functional dysregulation, cell-to-cell spread still occurs for gB_C507S mutated viruses as reflected by an increase in the focal CPE and the cell-associated viral DNA, yet the cell-free release is substantially reduced and non-infectious.

Intriguingly, the focal growth changed to a parental strain-like spread after a low-level replication (stagnation) period coinciding with the evolution of additional mutations in gB, which indicates an, at least partial, compensation of the gB fusion defect. Even more remarkable is the number of compensatory mutations mapping to gB DomV (Fig. 5) attributing an important functional relationship between DomIII and DomV. However, the proposed functional compensation is not due to physical proximity or cross-protomer contacts between C507 and the DomV mutation sites in the given gB trimer structures (Fig. 5C). Recent determination of the pre-fusion structure of HCMV gB shows that DomV, by contrast to DomIII, undergoes substantial rearrangement during the conformational change (1, 6). Refolding and extension of DomV are needed for the transition of the extended intermediate to the post-fusion conformation. DomV drags the membrane proximal region and transmembrane domain close to the fusion loops in DomI, bringing the two membranes together. In the post-fusion conformation, the three α helices of DomV (α5, α6, and α7) form an extensive interface for trimerization and/or potential interactions with the neighboring DomI and DomII (Fig. 5C). Based on the data from the *in vitro* cell–cell fusion assay, it appears that the second-site DomV mutations are structural mutations compensating for the C507S fusion defect as none of these mutations alone displays a higher fusogenicity than the wild-type gB construct. Hence, it can be speculated that the newly evolved mutations in DomV compensate for the engineered instabilization, which might be particularly disadvantageous in the last steps of the fusion process. Further *in vivo* studies with the individual DomV mutations may provide more detailed insights into their compensatory roles as the *in vitro* data have already shown different effects on gB fusogenicity.

The crucial role of DomV as a fusion regulator was previously shown for alphaherpesviruses (2). Point mutations in the DomV arm region, for example, cause a reduced fusion ability for HSV-1 (41), revertable by V705I and N709H in α7 (42). Furthermore, N735S in α7 of PrV (*Suid alphaherpesvirus 1*) reverts an entry-deficient mutant back to hyperfusogenicity (43). Here, we identified I683T homologous to V705I in HSV-1, which likewise reverts fusion-deficient gB_C507S (Fig. 5B). The variant allele Q/R699 corresponding to E665 in HCMV, localized in α6 of VZV, of which Q699, dominant in the live varicella Oka vaccine and a determinant for its attenuation, showed significantly lower fusion activity than R699 (46). In addition, we identified novel mutations localized within or in proximity of α6 and α7 that were critically involved in the regulation of fusion and spread of the betaherpesvirus HCMV (Fig. 5A through C). Together, these findings underline the key role of the proper rearrangement of the highly conserved DomV in regulating the fusogenic ability of gB not only in α- but also in β-herpesviruses. Furthermore, AD6 in DomV, the region where all our compensatory DomV mutations are found, seems to be an important target for a gB/MF59 vaccine-specific antibody response indicating its potential clinical importance (47).

Upon phenotype change and acquirement of additional mutations in gB_C507S, the virus release and the infectivity increased in relation to the abundance of the second-site mutations. Accordingly, the virions became infectious for both, fibroblasts and epithelial cells—with an epithelial-to-fibroblast ratio resembling the parental strain (39)—indicating a reversion of the mutant phenotype. However, the increase in release was not as fast as expected even after further propagation on fibroblasts, which suggests that the additional mutations are unable to completely rescue the mutant phenotype. Moreover, our deep sequencing data revealed that none of the second-site gB mutations reached near 100% dominance while the originally introduced C507S remained stable.

The finding that gB_C507S alone completely abolishes growth on epithelial cells in contrast to fibroblasts reflects the mechanistic differences in the fusion process. Virus entry into fibroblasts occurs under neutral pH at the plasma membrane and into epithelial cells under low pH at the endosomal membrane (13, 14). We assume that exposure to low pH further alters the impaired HCMV gB structure, as shown for HSV-1 gB (48), leading to these drastic effects on epithelial cells. Here, gB_C507S would underline this hypothesis due to the contrasting growth phenotypes in both cell types. Another not mutually exclusive explanation relies on the cell type-specific requirements of the distinct gH/gL complexes for gB triggering (12, 49, 50). Either trimer or pentamer can mediate virus spread on fibroblasts while pentamer is sufficient on epithelial cells (51–54). Hence, we hypothesize that gB_C507S affects the interaction with the pentamer stronger than with the trimer. Consequently, an in-depth analysis can provide a better understanding of the gB-mediated fusion triggers in both, cell types and complex interactions.

The polymorphic subunits of the core fusion machinery, in particular gB, gH, and gO (24), may also influence the fusion process. Here, we compared gB mutant phenotypes in three gO genotypic backgrounds, gO_GT1c_, gO_GT3_, and the chimera gO_GT1c3_ (36). gO_GT1c3_ virions carry a substantially lower content of trimer than gO_GT1c_ and gO_GT3_, while gO_GT3_ and gO_GT1c3_ infect epithelial cells better. We assumed that distinct growth properties may differentially contribute to the effects of gB mutations. Here, we were unable to see an obvious gO-dependent effect on mutant phenotypes. Moreover, the same second-site gB mutations emerged independently (E671K) or affected the same residue (Y667D/C), each in two different gO backgrounds (Table 2). In this study, we solely used one genotype for gB and gH. To further clarify whether or to what extent various combinations, as seen in HCMV clinical strains (29), influence gB-mediated fusion needs to be tested.

Inspection of all available HCMV gB sequences shows that Y494 and I495 are highly conserved while around 30% carry a serine instead of a glycine at site 493 (gB genotype 3). This polymorphism may explain why a substitution of this residue to proline has a less severe effect on the gB function. gB_G493P allows growth on both cell types whereas gB_Y494P and gB_I495P completely abolish virus growth pointing to a complete loss of gB function. Y494 maps to the homologous residues H516 and H527 in HSV-1 and VZV, respectively, where a proline substitution leads to functional arrest in the pre-fusion conformation (37, 38). Even though also gB_Y494P and gB_I495P were expressed *in vitro,* we need further investigations if their loss of function in HCMV is due to a pre-fusion arrest or due to folding, processing, or transport. In contrast, gB_G493P grew almost parental strain-like on fibroblasts and with severe growth retardation on epithelial cells. We assume that also a low pH and/or differences in the gB processing and/or the interaction with the different gH/gL complexes further weaken the gB function. gB_G493P lacks any emergence of second-site gB mutations, hinting at a weakened but still proper gB transition that cannot be structurally compensated by another mutation within the gB gene as it is assumed for gB_C507S.

Additional mutations at other genomic locations were sporadically observed in gB_C507S and gB_G493P on either cell type. Exceptionally, UL131A_T101S evolved with around 100% frequency in one gB_G493P clone in epithelial cells implying a growth advantage over the initial mutant (Fig. 2B). The associated formation of large dense foci may reflect a change in the mode of virus spread (Fig. S4B). UL131A, a highly conserved component of the pentamer (55, 56), plays a role in virus release (57, 58), and mutations thereof influence the mode of spread due to a change in the ratio of gH/gL complexes (18, 19). From our data, it appears that the emergence of T101S renders the viruses more efficient in cell-to-cell spread and virus release, which is well in line with the predicted functions of UL131A in receptor binding (49).

In summary, we have introduced single-point mutations situated in the DomIII α3 helix of HCMV gB and characterized the viral behavior in long-term evolutionary culturing. Our findings underline the functional importance of DomIII and DomV during the transition process on which future studies could focus to better understand the cell entry and especially the mode of spread in different cell types, important for vaccine and drug development.

## MATERIALS AND METHODS

### Cells and viruses

HFFs, ARPE-19 (ATCC, Manassas, VA), and human embryonic kidney cells (HEK293T) were cultured as previously described (36, 44). Reconstituted virus stocks stored at −80°C were further propagated on HFFs in T25 or T75 flasks until a CPE of >90%.

### Generation of gB mutants

Point mutations were individually introduced into BAC TB40-BAC4-luc DNA (39) carrying either gO GT1c, GT1c3, or GT3 sequence (36) via *en passant* mutagenesis (59) and purified for reconstitution as previously described (40). Sequence correctness prior to reconstitution was validated by WGS.

### Transfection, reconstitution, and long-term culturing

HFF and ARPE-19 cells were seeded on a six-well plate (1–1.5 × 10^5^ cells/mL) 24 h prior to the transfection. For transfection, 2.5-µg BAC-DNA for all mutants except for gB_G493P_gOGT1c_ clone 2 (2.0 µg) and gB_G493P_gOGT1c_ clone 1 (3.0 µg) were used in addition to 0.5 µg of CMV71 plasmid (kindly provided by Mark Stinski) encoding pp71 for increasing the efficiency of virus reconstitution, 12 µL Viafect Transfection Reagent (Promega). and MEM(Minimum Essential Medium) without antibiotics and FCS (Fetal Calf Serum) as previously described (36). Cells were passaged into T25 flasks after 10–13 days and T75 flasks after 23–26 days. ARPE-19 cells were additionally passaged into T25 flasks after 63 days. Medium change was performed up to twice a week. Two to four independent transfections were performed for each BAC mutant.

### Viral growth during reconstitution

CPE in cell culture monolayer was visualized using a 4× or 10× objective with the Leica MC170HD microscope and the Leica Application Suite X program every 3–4 days. Cells and supernatant were harvested at > 80% CPE in HFFs and at around 50% CPE in ARPE-19 cells. At each medium change, 2 µL of the supernatant was immediately transferred to 2 mL lysis buffer for DNA extraction using NucliSens EasyMag (BioMérieux) according to the manufacturer’s protocol, and HCMV-DNA was quantified (40). Residual 1-mL aliquots were stored at –80°C before being used for further infectivity testings. During each passaging step and at harvest, DNA was isolated from a 500 µL aliquot of the cell suspension for HCMV-DNA and genomic beta-globin DNA quantification (40). Another 300 µL of the cell suspension was used for the determination of RLUs by mixing equal parts of steady-Glo substrate (Promega) to 100 µL cell suspension, incubated for 5 min at RT (room temperature), and luminescence was measured with a Synergy HTX multimode reader (BioTek) in triplicates. Extracted DNA was stored at −20°C before being subjected to sequencing.

### Long-range gB PCR

Enrichment of the gB gene was performed by long-range PCR generating a 7,956-bp amplicon as previously described (60).

### Next-generation sequencing

Isolated BAC-DNA, total DNA, and long-range amplicons were used as input DNA for sequencing with paired-end reads (2 × 150–250) on the MiSeq platform using v2 reaction chemistry (Illumina). The sequencing reads were analyzed in CLC Genomics 21.0 (Qiagen) as previously described (61).

### Infectivity assay and immunofluorescence

A hundred microliters of cleared supernatants from all time points of medium change were added to HFF and ARPE-19 cells seeded in 96-well plates (1–1.5 × 10^5^ cells/mL) with one additional 1:2 dilution and incubated for 48 h (HFF) and 72 h (ARPE-19) at 37°C and 5% CO_2_. Then, the medium was removed, and cells were washed once with 1× phosphate-buffered saline (PBS) and lysed with 100-µL Glo Lysis Buffer (Promega) to perform a luciferase assay as described above. Results were shown in log_10_ RLU. For immediate early staining of C507S mutants, cell-free viruses harvested prior to and after the phenotype change were adsorbed to HFFs for 2 h at 10°C under 800 × *g* centrifugation. After two cold PBS washes, cells were treated with 44% polyethylene glycol (PEG) (pre-warmed to 37°C) for 30 s, followed by 10 washes with a warm culture medium to remove the PEG. The cultures were incubated at 37°C until the characteristic phenotypes were observed to perform immunofluorescence analysis. PEG 6000 (Fluka) was prepared as a 60% (wt/wt) solution in PBS and diluted with PBS to 44%.

### Determination of encapsidated virus particles

Stored HFF-derived virus stocks were thawed and in parallel either treated with TurboDNase (Thermo Fisher) to determine the encapsidated HCMV genome copy numbers or used for infectivity testing by a luciferase assay. For copy number determination, 100 µL of master mix [73 µL H_2_O, 20 µL 10× DNase buffer, 5 µL 10× PBS, and 2 µL TurboDNase (2 units/µL)] were added to 100 µL of the virus sample and incubated for 1 h at 37°C at 1,400 rpm. Immediately thereafter, DNA was extracted and HCMV-DNA was quantified (40). All experiments were performed in technical duplicates.

### Generation of gB expression constructs

Point mutations were introduced into the codon-optimized TB40gB-VSV-G expression constructs (9) by site-directed mutagenesis (Life Technologies). The primers are listed in Table S2.

### Cell–cell fusion assay

The DSP cell membrane fusion assay was described before (44). HEK293T-DSP-mix cells were transfected with the unmutated and mutated forms of fusion-competent TBgB/VSV-G through calcium phosphate precipitation. After 1–3 days, cell–cell fusion was monitored by GFP fluorescence using a Leica TCS SP5 confocal microscope or quantified by bioluminescence upon cleavage of the cell-permeable substrate EnduRen (Promega).

## Data Availability

Raw sequence data have been deposited in NCBI Sequence Read Archive under BioProject ID PRJNA1156641. We will make other data fully available and without restrictions.

## SUPPLEMENTAL MATERIAL

Fig. S1 mbio.01812-24-s0001.tif Viral replication differences among gB mutants in TB40gOGT3 background.

Fig. S2 mbio.01812-24-s0002.tif Significant differences in release of viral DNA between parental strain and gB_C507S mutants after transfection.

Fig. S3 mbio.01812-24-s0003.tif Infection efficiency of epithelial cell-derived viruses.

Fig. S4 mbio.01812-24-s0004.tif Spread morphology of gB_gOGT1c and gB_gOGT1c3 mutants on fibroblasts and epithelial cells.

Fig. S5 mbio.01812-24-s0005.tif Spread morphology of gB_gOGT3 mutants.

Legends mbio.01812-24-s0006.docx Legends to the supplemental figures.

Supplemental Tables mbio.01812-24-s0007.docx Tables S1 and S2.

ASM does not own the copyrights to Supplemental Material that may be linked to, or accessed through, an article. The authors have granted ASM a non-exclusive, world-wide license to publish the Supplemental Material files. Please contact the corresponding author directly for reuse.
