# Supplementary figures and images for: Growth defect of domain III glycoprotein B mutants of human cytomegalovirus reverted by compensatory mutations co-localizing in post-fusion conformation
Source: mBio. 2024 Sep 24;15(10):e01812-24. doi: 10.1128/mbio.01812-24 (PMC11481916; doi:10.1128/mbio.01812-24)

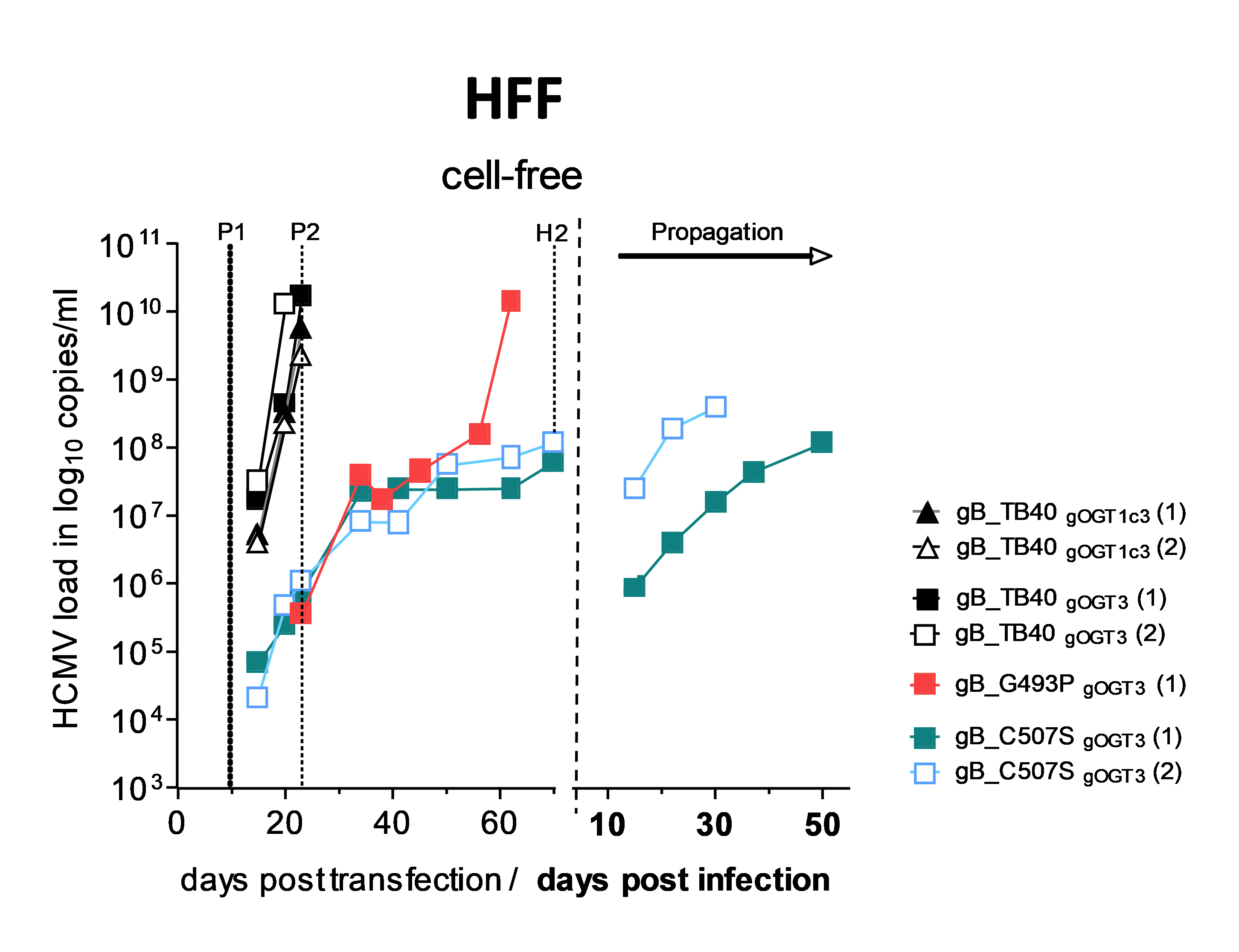

Supplement: Fig. S1 — Viral replication differences among gB mutants in TB40gOGT3 background. [file mbio.01812-24-s0001.tif]

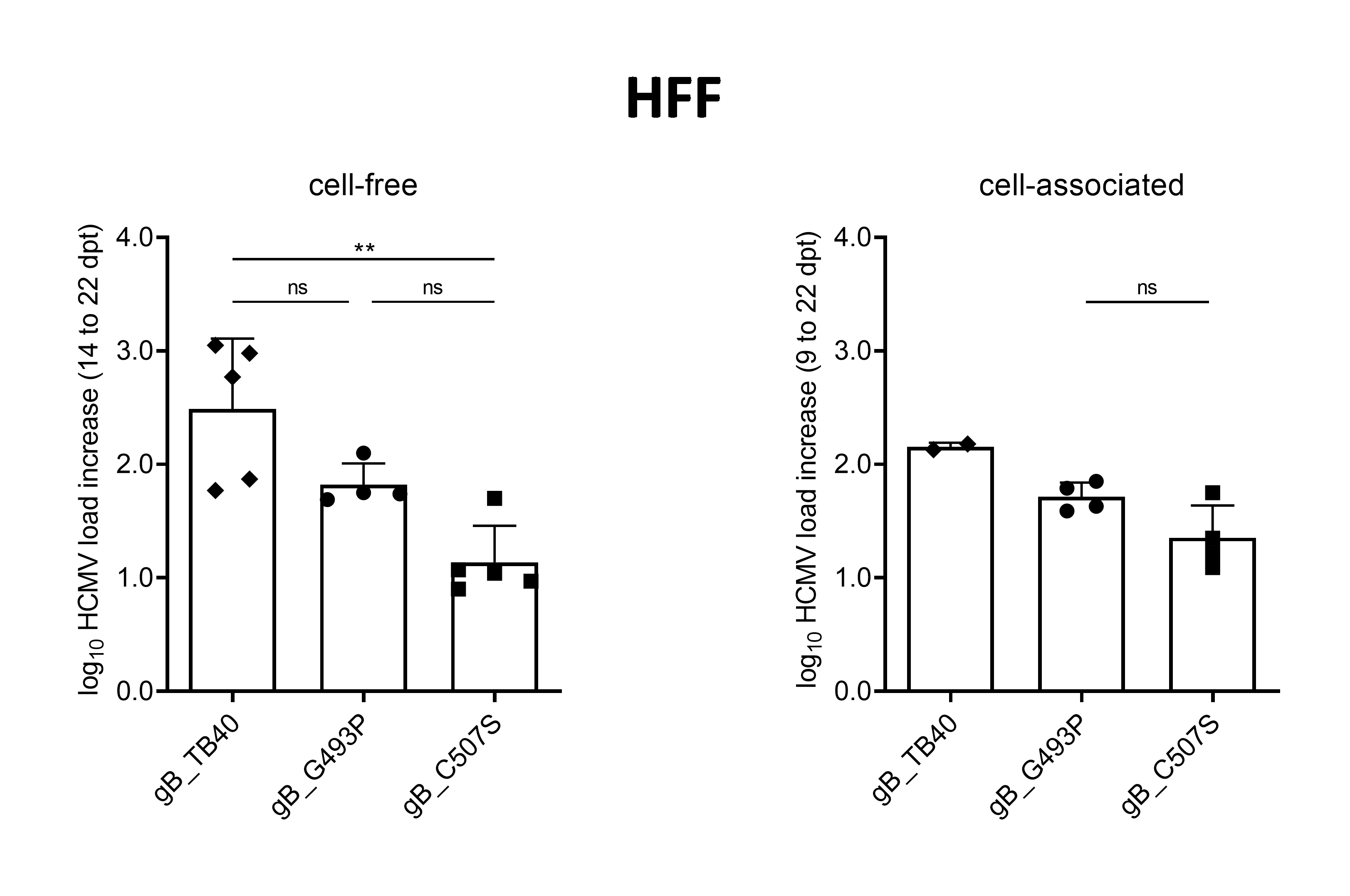

Supplement: Fig. S2 — Significant differences in release of viral DNA between parental strain and gB_C507S mutants after transfection. [file mbio.01812-24-s0002.tif]

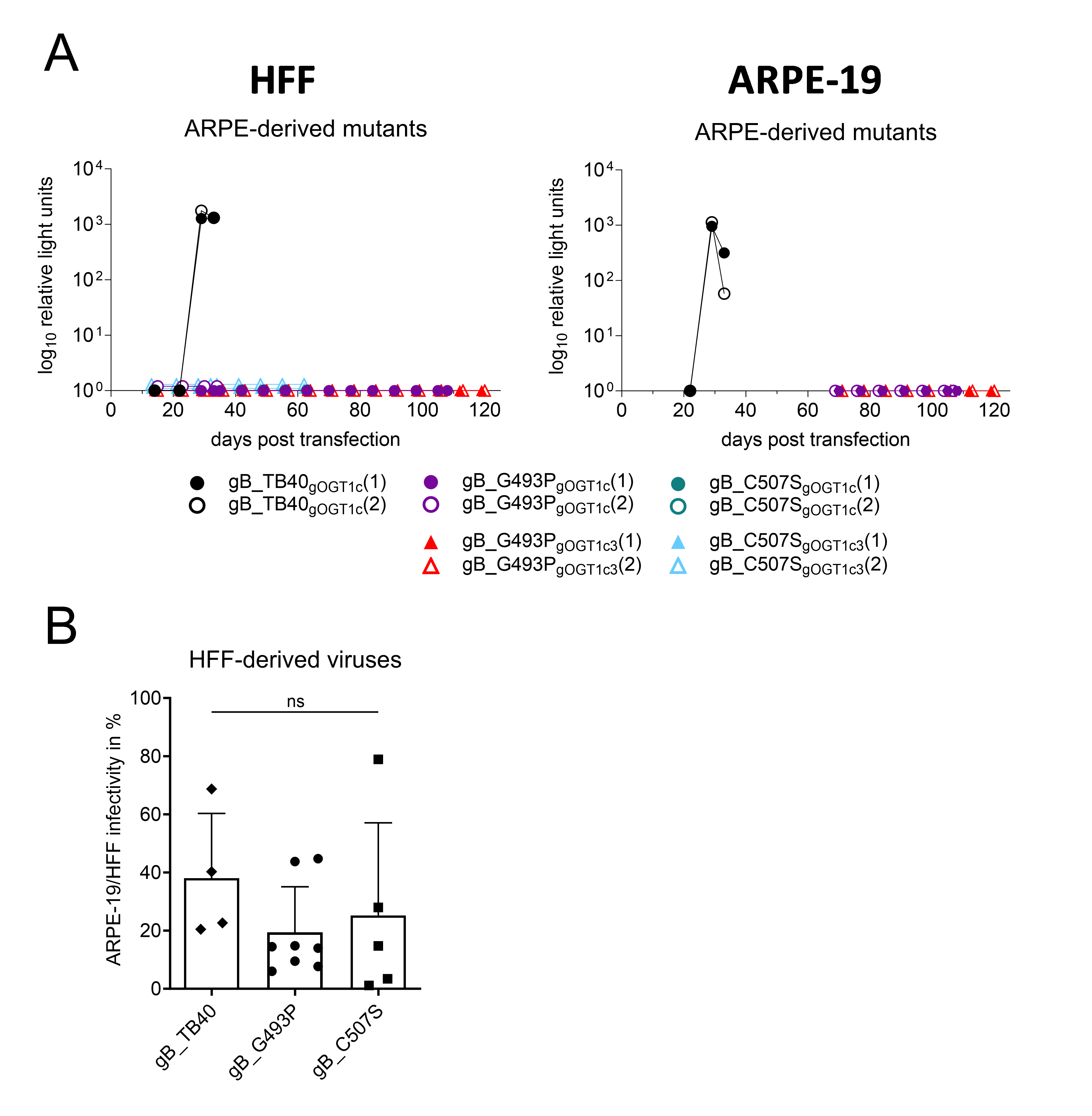

Supplement: Fig. S3 — Infection efficiency of epithelial cell-derived viruses. [file mbio.01812-24-s0003.tif]

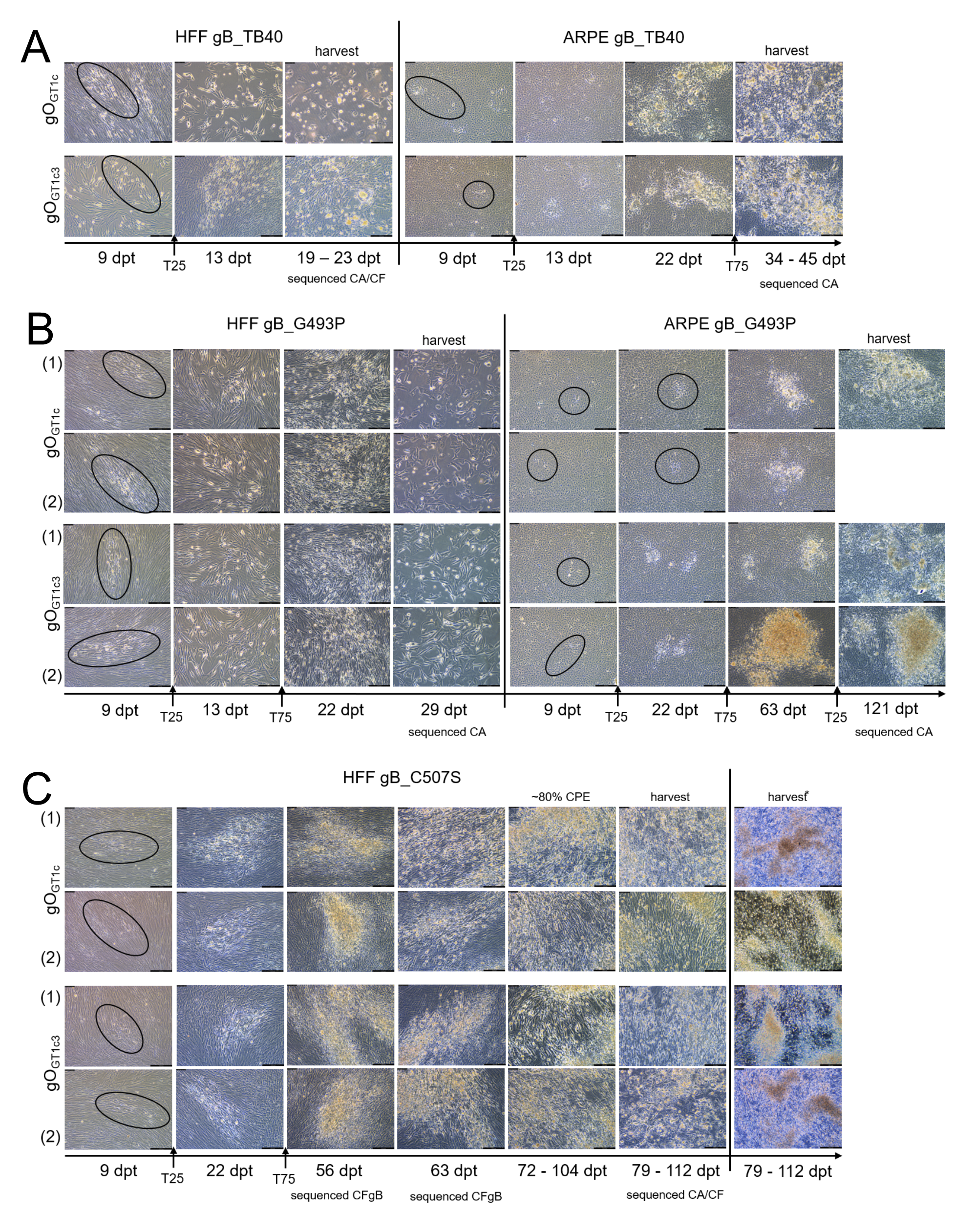

Supplement: Fig. S4 — Spread morphology of gB_gOGT1c and gB_gOGT1c3 mutants on fibroblasts and epithelial cells. [file mbio.01812-24-s0004.tif]

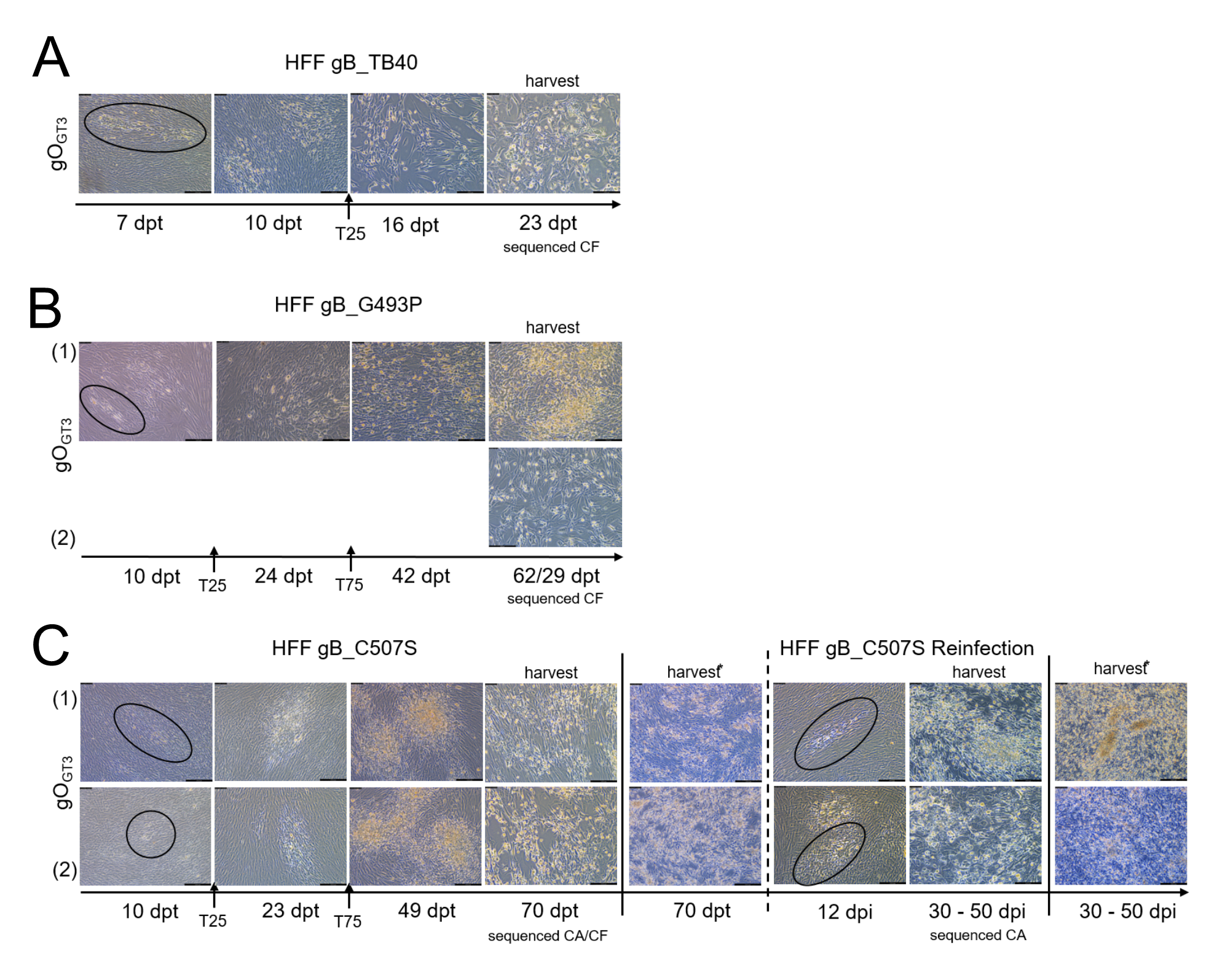

Supplement: Fig. S5 — Spread morphology of gB_gOGT3 mutants. [file mbio.01812-24-s0005.tif]
