# Supplementary material for: Growth defect of domain III glycoprotein B mutants of human cytomegalovirus reverted by compensatory mutations co-localizing in post-fusion conformation
Source: mBio. 2024 Sep 24;15(10):e01812-24. doi: 10.1128/mbio.01812-24 (PMC11481916; doi:10.1128/mbio.01812-24)
Supplement: Legends — to the supplemental figures. [file mbio.01812-24-s0006.docx]

**Supplementary Figure Legends**

**Supplementary Figure 1. Viral replication differences among gB mutants in TB40gOGT3 background.** Fibroblasts (HFF) were transfected with the respective TB40-BAC-luc DNA and long-term cultured. Harvested cell-free samples of both clones of gB_C507S_gOGT3_ (H2) were further propagated on HFFs for 30 – 50 days post infection. After transfection and infection, cell-free samples were taken from the supernatant at the indicated time points and subjected to viral DNA load determination. During reconstitution all clones were transferred from a 6-well plate to a T25 flask at 9 dpt (P1) and the gB mutants were further passaged into a T75 flask after 22 dpt (P2). HCMV, human cytomegalovirus; HFF, human foreskin fibroblast; GT, genotype.

**Supplementary Figure 2. Significant differences in release of viral DNA between parental strain and gB_C507S mutants after transfection.** The log_10_ increase in viral DNA load between 14 and 22 dpt and 9 and 22 dpt in cell-free and cell-associated samples, respectively, was calculated for all gB mutants. Mutants with the same gB mutation independent of the gO genotype (GT) background were grouped. Mean values with error bars indicate standard deviation (SD). **, p < 0.01; ns, not significant, one-way ANOVA with Tukey's multiple comparisons test; HCMV, human cytomegalovirus; HFF, human foreskin fibroblast; dpt, days post transfection; GT, genotype.

**Supplementary Figure 3. Infection efficiency of epithelial cell-derived viruses.** A) Infection efficiency of ARPE-derived viruses collected as cell-free viruses from the supernatant during the post-transfection period was determined. After infection, fibroblasts (HFF) were incubated for 48 h and ARPE-19 cells for 72 h before determination of relative light units (RLUs) in the cell lysates as the read out of infection efficiency. HFF, human foreskin fibroblast; ARPE-19, retinal pigment epithelial cells; RLU, relative light units.

**Supplementary Figure 4. Spread morphology of gB_gOGT1c and gB_gOGT1c3 mutants on fibroblasts and epithelial cells.** Human foreskin fibroblasts (HFF) or epithelial cells (ARPE-19) were transfected with the respective TB40-BAC-luc DNA and long-term cultured. After transfection, pictures were taken every three to four days via light microscopy (Leica). Representative fields are shown at the indicated time points in 10X if not otherwise indicated. A) Parental strains gB_TB40gOGT1c (top) and gB_TB40gOGT1c3 (bottom) in HFF (left) and in ARPE-19 cells (right). Cell-free (CF) and cell-associated (CA) samples were sequenced at the time of harvest by whole genome sequencing (WGS). B) gB_G493P mutants in HFF (left) and ARPE-19 cells (right). WGS of CA samples at the time of harvest. C) gB_C507S mutants in HFFs. CA and CF samples were WGS sequenced at the time of harvest. CF samples at 55 and 62 days post-transfection (dpt) were enriched through long range PCR for gB (CFgB) prior to sequencing due to a low viral load. Black circles indicate cytopathic effects (CPE). HCMV, human cytomegalovirus; HFF, human foreskin fibroblast; ARPE-19, retinal pigment epithelial cells; T25, transfer into a T25 flask; T75, transfer into a T75 flask; (1), clone 1; (2), clone 2; dpt, days post transfection; CF, cell-free; CA, cell-associated; CPE, cytopathic effect; CFgB, cell-free gB.

**Supplementary Figure 5. Spread morphology of gB_gOGT3 mutants.** Human fibroblasts (HFF) were transfected with the respective TB40-BAC-luc DNA and long-term cultured. After transfection, pictures were taken every three to four days via light microscopy (Leica). Representative fields of each picture are shown at the indicated time points in 10X if not otherwise indicated. A) gB_TB40gOGT3parental strain. Cell-free (CF) samples were sequenced at the time of harvest by whole genome sequencing (WGS). B) gB_G493P mutants. CF samples were WGS sequenced at the time of harvest. C) gB_C507S mutants. WGS of cell-associated (CA) and CF samples at time point of harvest with ~ 80% cytopathic effect (CPE). HFFs were subsequently reinfected with gB_C507S CF samples from the time of harvest for further culturing (right) until 30 - 50 days post infection (dpi). CA samples were WGS sequenced at the time of harvest. HCMV, human cytomegalovirus; HFF, human foreskin fibroblast; T25, transfer into a T25 flask; T75, transfer into a T75 flask; (1), clone 1; (2), clone 2; dpt, days post transfection; dpi, days post infection; CF, cell-free; CA, cell-associated; CPE, cytopathic effect.
